# Epigenetic modifications: Allusive clues of lncRNA functions in plants

**DOI:** 10.1016/j.csbj.2023.03.008

**Published:** 2023-03-11

**Authors:** Wenjing Yang, Quanzi Bai, Yan Li, Jianghua Chen, Changning Liu

**Affiliations:** a CAS Key Laboratory of Tropical Plant Resources and Sustainable Use, Yunnan Key Laboratory of Crop Wild Relatives Omics, Xishuangbanna Tropical Botanical Garden, Chinese Academy of Sciences, Kunming, 650223, China; b University of Chinese Academy of Sciences, Beijing 100049, China; c Center of Economic Botany, Core Botanical Gardens, Chinese Academy of Sciences, Menglun, Mengla, China

**Keywords:** LncRNA, Epigenetic regulation, Epigenomes, Plant

## Abstract

Long non-coding RNAs (lncRNAs) have been verified as flexible and important factors in various biological processes of multicellular eukaryotes, including plants. The respective intricate crosstalk among multiple epigenetic modifications has been examined to some extent. However, only a small proportion of lncRNAs has been functionally well characterized. Moreover, the relationship between lncRNAs and other epigenetic modifications has not been systematically studied. In this mini-review, we briefly summarize the representative biological functions of lncRNAs in developmental programs and environmental responses in plants. In addition, we particularly discuss the intimate relationship between lncRNAs and other epigenetic modifications, and we outline the underlying avenues and challenges for future research on plant lncRNAs.

## 1 Introduction

To efficiently adapt to the habitat environment, plants have evolved intricate strategies to orchestrate temporal and spatial gene expression patterns in response to exogenous environmental signals and endogenous developmental cues. Among these precise and complex strategies, epigenetic regulation mechanisms which mainly including DNA methylation, histone modification, histone variant, chromatin remodeling and noncoding RNAs [1], play a dispensable role. Due to the vital function in solving global challenges such as crop yield and food security, epigenetic study in plants has become the forefront and hotspots during the past decades [2], [3].

Long non-coding RNAs (lncRNAs) are classified as a type of ncRNAs whose length is more than 200 nt and do not encode proteins or have extremely low encoding capacity [4]. At the early stage of their discovery, lncRNAs were considered "noise" of genome transcription without discernible biological function. However, lncRNAs are abundant and widely distributed in eukaryotes, and they exhibit important roles in the biological process of animals and plants. They were involved in X chromosome silencing, genomic imprinting, chromatin modification, transcriptional activation transcription interference, and other regulatory processes [5], [6], [7], [8].

During the past decade, an increasing number of studies demonstrated the vital roles of plant lncRNA in multiple biological processes [9], [10], [11], [12]. However, the internal relationships between lncRNAs and other epigenetic modifiers in plants remain elusive, including how the lncRNA transcript levels are regulated by other epigenetic factors, and how lncRNAs cooperate with other epigenetic factors to function in gene transcriptional regulation. In this mini-review, we summarize the participation of lncRNAs in developmental programs orchestration and environment responses, and we mainly discuss the interactions between multiple epigenetic factors and lncRNAs in plants, aiming at obtaining clues with guard to lncRNA functions and regulatory mechanisms.

## 2 Landscapes of epigenomic data source in plants

Epigenetics participate in nearly all developmental processes of plants, from seed germination to flowering followed by pollination and seed ripening, and also respond to various environmental cues [10]. In *Arabidopsis*, epigenetic modification at the *FLOWERING LOCUS C* (*FLC*) locus through H3K27me3, H2Bub and lncRNAs play vital roles with respect to flowering time regulation [13], [14], [15], [16], and the key chromatin modifications including DNA, RNA, and histone methylation or acetylation are indispensable in the light signaling pathway [17]. In rice, studies on various genome-wide epigenetic signals identified epigenomic variations that are significantly associated with plant growth, fitness, yield and other important agronomic traits [18]. Until now, high throughput sequence technologies have depicted the multidimensional epigenome landscape in various plants, thus providing rich data sources for further epigenetic studies.

As shown in Table 1, six databases have collected and organized more than ten thousands of public datasets including histone modification (ChIP-seq), DNA methylation on 5-methylcytosine and *N*^6^-methyladenine (BS-seq, meDIP-seq, SMRT-seq), and chromatin states (ATAC-seq, DNase-seq, MNase-seq and FAIRE-seq) [19], [20], [21], [22], [23], [24]. In these databases, model plants such as *Arabidopsis thaliana* (*A. thaliana*), *Oryza sativa* (*O. sativa*) and *Zea mays* (*Z. mays*) account for the highest proportion of datasets, especially the data source in *O. sativa* is most prevalent. Access to such data is vital for biological researchers to visibly gather detailed information on specific target genes. For example, the peak enrichment distribution of histone modifications can be easily searched through a genome browser tool based on web service. In epigenetic databases, gene annotation information with regard to epigenetic modifications in plants is nearly comprehensive for protein coding genes. Although the epigenetic datasets can be obtained from PlantDB V2.0, which is a plant lncRNA database [25], the number of datasets (454 datasets across seven species) pertaining to epigenetics in plant lncRNA databases was much fewer than that summarized in databases (Table 1). Therefore, combined with the support of public data sources, especially for epigenetic modification analysis, further exploring the biological roles of lncRNAs related to epigenetic modifications seems to be practical and meaningful.

**Table 1:** The data source of plant epigenomic landscapes.

Database	Species	Epigenomic types			Datasets	References
		Histone modifications	DNA methylation	chromatin states		
PlantDHS	*A. thaliana*, *B. distachyon*, *O. sativa*	ChIP-Seq		DNase-Seq	23	[19]
PCSD	*A.thaliana*, *O. sativa* and *Z. mays*	ChIP-seq	meDIP-seq	DNase-seq, MNase-seq	100	[20]
MethBank	*O. sativa*, *G.max*, *M. esculenta*, *P. vulgaris*, *S. lycopersicum, G. max*		BS-seq		336	[21]
eRice	*O. sativa subsp. Japonica* and *indica*	ChIP-seq	SMRT-seq	DNase-seq	124	[22]
RiceENCODE	*O. sativa*	ChIP-seq	BS-seq	ATAC-seq, MNase-seq, FAIRE-seq	972	[23]
ChIP-Hub	*A. thaliana*, *O. sativa etc*. (>40)	ChIP-seq		ATAC-seq, DNase-seq	> 10,000	[24]

*A. *thaliana*: *Arabidopsis thaliana*, *B*. *distachyon*: *Brachypodium distachyon*, *O*. *sativa*: *Oryza sativa*, *Z*. *mays*: *Zea mays*, *M*. *esculenta*: *Manihot esculenta*, *S*. *lycopersicum*: *Solanum lycopersicum*, *G*. *max*: *Glycine max*, *P*. *vulgaris*: *Phaseolus vulgaris.*

## 3 The biological roles of lncRNA in plants

LncRNAs, which are transcribed by various RNA polymerases, including RNA polymerase II-V [26], are involved in intricate biological processes, as shown through multiple approaches. Most transcribed lncRNAs occur very low abundances in plants, however they have been verified to function in diverse developmental processes and environmental responses [27]. In model plants including *A. thaliana*, *Solanum lycopersicum* (*S. lycopersicum*), *Glycine max* (*G. max*), *Medicago truncatula* (*M. truncatula*), *O. sativa*, *Z. mays* and *Triticum aestivum* (*T. aestivum*), lncRNAs are involved in organ morphogenesis [28], [29], [30], seedling photomorphogenesis [31], flowering [14], [32], [33], male sterility [34], [35], seed germination [36] and fruit ripening [37]. Throughout their life cycle, plants may encounter multiple abiotic stresses (resulting in salt, drought, extreme temperature and nutrient stress) and biotic stresses (including bacteria, fungi, viruses and pests invasion). With regard to these fluctuated environmental stimuli, the vital roles of lncRNAs in response to drought and salt stresses [38], [39], extreme temperature stress [40], nutrient stress [41], [42], and pathogen stress [43], [44] have been confirmed.

Among those complex biological processes, a small part of lncRNAs have been well characterized, which flexibly participated in regulating target genes as cis or trans-acting elements by interacting with DNA, RNA, or proteins [10], [45]. In common, lncRNAs participate in regulatory mechanisms refer to neighboring and distant gene transcription, RNA splicing and stability, and as miRNA sponges [45]. Apart from these regulatory mechanisms, the intricate and precise cooperation with epigenetic modifiers to orchestrate gene transcription or chromatin structure is worth future research in plants.

## 4 Survey of functional lncRNAs associated with epigenetic modifications

**Fig. 1:**
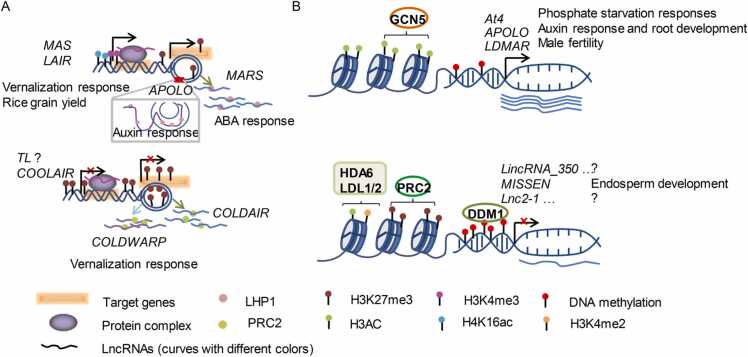
A summarized work model for functions of lncRNAs associated with epigenetics modifiers in plants. LncRNAs coordinate regulation with epigenetic modifier to activate target genes (eg. *MAS*, *LAIR*, *MARS* and *APOLO*, shown in the upper part of the diagram A), and repress target loci (e.g., *COOLAIR*, *COLDAIR*, *COLDWARP* and *TL*, shown in the lower part of the diagram A). Additionally, lncRNAs are positively transcribed by epigenetic activation marker enrichment or suppression marker removal (such as *At4*, *APOLO* and *LDMAR*, shown in the upper part of the diagram B), and repress by increase of suppression marker or decrease of activation marker by epigenetic modifiers (such as *LincRNA_350*, *MISSEN* and *Lnc2–1*, shown in the lower part of the diagram B).

In the past ten years, the functions of several lncRNAs in plants have been well characterized. Here, we review, in particular, the lncRNAs associated with epigenetics marks to alter target gene transcription (Table 2). According to their regulatory relationship with other epigenetic modifiers, lncRNAs are classified into two categories (Table 2), i.e., lncRNAs that actively cooperate with epigenetic modifiers to trigger downstream targets (Fig. 1A), and lncRNAs the transcription of which is controlled by epigenetic modifiers (Fig. 1B).

**Table 2:** Regulation of lncRNA associated with epigenetics modifiers in plants.

	LncRNA	Type	Function	Molecular mechanism	References
Coordinated regulation with epigenetic modifier	*COOLAIR*	NAT-lncRNA	FLC gene silencing during vernalization	histone methylation	[14], [16]
	*COLDAIR*	incRNA		histone methylation, chromatin loop	[32]
	*COLDWRAP*	lincRNA		histone methylation, chromatin loop	[33]
	*APOLO*	lincRNA	response to auxin	chromatin loop, histone methylation and DNA methylation	[28], [50], [51]
	*MARS*	lincRNA	ABA response	histone methylation and chromatin loop	[49]
	*MAS*	NAT-lncRNA	vernalization response	histone methylation	[46]
	*LAIR*	NAT-lncRNA	rice grain yield	hitstone methylation and acetylation	[47]
	*TL*	NAT-lncRNA	leaf morphological development	histone methylation	[48]
Passive regulation by epigenetic modifier	*APOLO*	lincRNA	response to auxin and root development	DNA demathylation	[50]
	*LDMAR*	lincRNA	photoperiod-sensitive male sterility	RNA-dependent DNA methylation	[34], [52]
	*Lnc2–1/Lnc3–3/Lnc4–1/* *Lnc6–1/Lnc8–1/Lnc12–1*	lincRNA	unkonwn	DNA methylation	[59]
	*At4*	lincRNA	phosphate starvation responses	histone acetylation	[53]
	*LincRNA_350/LincRNA_351/* *LincRNA_470/LincRNA_700*	lincRNA	unkonwn	histone deacetylation and demethylation	[54]
	*MISSEN*	lincRNA	early endosperm development	histone methylation	[56]

* NAT-lncRNA: natural antisense transcript; incRNA: intronic RNA; lincRNA: long intergenic noncoding RNA.

During vernalization in *A. thaliana*, activation of three lncRNAs (*COOLAIR*, *COLDAIR*, and *COLADWARP*) is conducive to the suppression of *FLC* through enrichment of H3K27me3 at the *FLC* locus (Fig. 1A, lower part) [14], [16], [32], [33]. *COLDAIR* (transcribed from the first intron of *FLC*) and *COLDWRAP* (transcribed from the promoter region of *FLC*) cooperate to form a chromatin loop through directly binding to the PRC2 complex to establish a repression state of *FLC* locus [32], [33]. A further mediated precocious flowering lncRNA in *A. thaliana* termed *MAS* which is produced from the anti-sense strand of *MADS AFFECTING FLOWERING4* (*MAF4*) locus, activate *MAF4* transcription by recruiting WDR5a to enhance H3K4me3 level of *MAF4* loci [46]. Meanwhile, *LAIR* also an NAT-lncRNA, originated from *LRK* (encoding leucine-rich repeat receptor kinase), positively regulate grain yield in rice through binding OsMOF and OsWDR5 to the LRK1 gene region resulting in up-regulating its expression through the enrichment of H3K4me3 and H4K16ac [47]. Although *MAS* and *LAIR* (Fig. 1A, upper part) are involved in different biological processes, both of them are transcribed from the anti-sense strand of target gene loci and recruit homologs of histone-modifying enzymes to regulate target gene. In leaf development, *TWISTED LEAF* (*TL*) constrains its sense gene expression by mediating chromatin modifications [48]; however, the cooperators facilitating diverse histone modification level changes remain to be identified.

Besides, with regard to hormone response, the functions of two intergenic members, *MARneral Silencing* (*MARS*) and *APOLO* (Fig. 1A. upper part), are worthwhile noting. Through decoy of the LIKE HETEROCHROMATIN PROTEIN 1 (LHP1) protein, *MARS* orchestrates the H3K27me3 distribution and promotes the chromatin loop formation [49]. Coincidentally, *APOLO* also shows a close relationship with LHP1. *APOLO* interacted with LHP1 to promote the formation of the chromatin loop by APOLO-LHP1 in auxin response [28]. The formation of DNA–RNA duplexes (termed R-loops) is modulated by *APOLO* to target associated or distant loci [50]. Beyond those mentioned above, *APOLO* is also shown to coordinate VARIANT IN METHYLATION 1 (VIM1) to form *APOLO*-LHP1-VIM1 complex, directly regulating the transcription of the auxin biosynthesis gene *YUCCA2*. Interestingly, compared to *APOLO*, the sequence of lncRNA *UHRF1* in humans showed poor similarity but displayed similar performance in transcription regulation of *YUCCA2* [51], predicting that analysis of conservative regulatory mechanisms may be a feasible approach to study the conservation of lncRNAs. The coordination regulation to target loci between lncRNAs and histone modifiers reflects the ability of lncRNAs to affect gene regulation through recruiting, decoy mechanisms, and interaction with histone modifiers.

Reduced DNA methylation levels at the *APOLO* locus are also conducive to auxin-induced *APOLO* expression [50]. It is unclear how DNA methylation was removed during *APOLO* activation, however, an lncRNA was verified as downstream of epigenetics modifications (Table 2). In photoperiod-sensitive male sterility rice (Nongken 58 S), *Psi-LDMAR* (a siRNA) mediates methylation of the lncRNA *LDMAR* promoter region through the RdDM pathway to inhibit the transcription of *LDMAR*. Reduced *LDMAR* transcription leads to male sterility under long-day conditions [34], [52]. DNA methylation markedly affects lncRNA transcription; however, changes in histone methylation and acetylation level also affect lncRNA activity (Fig. 1B, upper part). In phosphate starvation responses, the lncRNA *At4* is directly targeted by histone acetyltransferase GCN5 mediated H3K9/14 acetylation [53]. The expression of a specific fraction of lincRNAs was possibly negatively regulated by HISTONE DEACETYLASE 6 (HDA6) and LSD1-LIKE 1/2 (LDL1/2). The enhanced level of H3Ac and H3K4me2 at increased expression lncRNA sites in *hda6* or *hda6/ldl1/2* mutant indicated that HDA6, LDL1, and LDL2 were potential regulators [54] suggesting different histone modifications may exhibit crosstalk and together target the same lncRNAs (Fig. 1B, lower part). In rice, numerous lncRNAs are more likely to be targets of repression by PRC2 rather than participate in regulation via PRC2 as they display high expression levels in PRC2 mutant [55]. Later, the lncRNA *MISSEN* was cloned because of a low-fertility phenotype after T-DNA insertion in rice, and a further study showed that its transcription was inhibited by H3K27me3 modification after pollination. After derivation and verification, *MISSEN* was up-regulated in the *emf2a* mutant [56], implying EMF2a is an upstream repressor in endosperm development. *MISSEN* is a good example, showing it is practical to research lncRNA functions according to transcriptome analysis of epigenetic modifier mutant to predict possible regulated lncRNAs, and based on phenotypes induced by lncRNA mutation to guess upstream potential regulator.

Although lncRNAs function in various biological pathways, the characteristics of low expression, poor sequence conservation and flexible roles render them elusive with regard to functional performance. Thus bioinformatic analyses of publicly available data are vital to speculate and examine how lncRNAs may work. Constant attention on such big data analysis across various plant species find that the hallmarks of histone modifications or DNA methylation mainly reflect effects at protein coding gene sites, while a considerable proportion of those located in non-coding regions cannot be ignored. In *A. thaliana*, analysis of large-scale ChIP-seq datasets produced the typical enrichment profile of various histone marks at the lncRNA loci (excluding some short regions near transcription start and termination sites), which disturbed similar to protein coding genes region [57], [58]. Among those histone marks, the expression of lncRNAs was preferentially correlated with H3K4me3, H3AC, H3K4me2/3 and H3K36me3 rather than with H3K9me2 and H3K27me3 [54]. Moreover, the expression level of a group of lincRNAs (such as *Lnc2–1*, *Lnc3–3*, *Lnc4–1*, *Lnc6–1*, *Lnc8–1*, and *Lnc12–1*) is sharply increased in *ddm1a/1b* mutant [59]. These lncRNAs are negatively regulated by chromatin-remodeling factor DECREASE IN DNA METHYLATION1 (DDM1) (Fig. 1B, lower part). In addition, absence of mCG in DNA methylation mutants has more impact on lincRNA transcription, compared to non-CG methylation in *A. thaliana*, *O. sativa*, and *S. lycopersicum* [60]. Hence, epigenetic modifiers universally determine transcription of protein coding genes, but their possibilities to affect transcription of lncRNA regions seem to be concerned.

In summary, the strong relationships between lncRNA transcription and epigenetic changes, no matter coordinated regulation or passive regulation mainly via writers or erasers of DNA methylation and histone modification (Fig. 1), imply lncRNAs may work in epigenetic modifiers mediated biological pathways.

## 5 Challenges of lncRNA functions digging from an epigenetic perspective

LncRNAs are widely involved in the development processes, and hormone and stress response in plants via co-transcriptional processes with multiple epigenetic factors. In contrast, these epigenetic modifiers can also control the activity of lncRNAs (Fig. 1, Table 2). LncRNAs are closely associated with epigenetic modifier mediated biological processes, and they could also be involved in small RNA (sRNA) regulation. For example, the activity of *LDMAR* depended on phasiRNA mediated DNA demethylation in rice [34], [52] suggesting lncRNA could also be targets of sRNAs. In addition, lncRNA could be precursors of sRNA (siRNAs and miRNAs) and modulate the transcription of downstream genes via controlling the production of sRNAs [61], [62]. Therefore, the relationships of lncRNAs, sRNAs and epigenetic modifiers probably modulate co-target loci in some highly organized and precise manners, which remain to be investigated. Those above speculations highlight the complexity, diversity, and challenges of regulatory mechanisms mediated by lncRNAs.

Meanwhile, similar to protein coding transcripts, lncRNAs could be modified with *N6*-methyladenosine and later exhibit close crosstalk with different epigenetic modification processes including writing or erasing of DNA methylation and histone modifications, which has been well informed in animals and humans but is worth to be explored in plants [63], [64]. Considering the lack of lncRNA sequence similarity, the identification of homologous lncRNAs across species as done for protein coding genes is currently unfeasible. This aspect thus warrants the hidden characteristics of lncRNAs and bias of lncRNAs function research in human or animals need to be distinguished for plant biologists.

Additionally, many kinds of integrated databases containing lncRNA annotation and function prediction data have been well developed for animals and humans [65], [66]. For example, the database Lnc2Meth specializes in providing services on regulatory relationships between lncRNAs and DNA methylation in various human diseases [67]. However, such comprehensive and detailed databases for plant lncRNA research are rather lacking. Considerable detailed work on data integration need to be done for lncRNA annotations, especially considering lncRNA related agronomic traits, which has been comprehensively conducted in rice [68]. Compared to animals and humans, some new high-throughput sequencing technologies for lncRNA annotation and function prediction have not been widely applied to plants. For instance, capture long-read sequencing, CAGE-seq, long-read RNA-seq, and RACE-Seq are useful for full-length of lncRNA annotation [69], [70], [71], and RIP-seq and CHIRP-seq are helpful for lncRNA-protein or lncRNA-chromatin interaction perdition [72], [73]. Nevertheless, some of these technologies above have only been started to be used in research on lncRNAs of cotton, rice and Arabidopsis [46], [58], [74]. Surprisingly, single molecule-based RNA structure sequencing was designed to capture single RNA molecule structure *in vivo* [75]. Further, the hyper-variable region of *COOLAIR* had stronger ability to interact with chromatin mediated slicing of *FLC* in response to cold and warm conditions in *A. thaliana* [75], confirming that various structures of the lncRNA isoforms could precisely modulate gene transcription. Moreover, algorithms or tools for lncRNA characteristics research, such as lncRNA coding potential and structure prediction are generally developed based on animal or human models [76]. Whether those perform well in plants remain to be confirmed. Consequently, further embed regulatory networks for lncRNA and epigenetic modifications will be discovered in plants with the emergence of respective methods and technologies.

## CRediT authorship contribution statement

**Wenjing Yang**: Conceptualization, Data curation, Investigation, Writing – original draft, Writing – review & editing. **Quanzi Bai**: Data curation, Investigation, Writing – original draft. **Yan Li**: Investigation. **Jianghua Chen**: Writing – review & editing. **Changning Liu**: Conceptualization, Funding acquisition, Supervision, Writing – original draft, Writing – review & editing.

## Declaration of Competing Interest

The authors declare that they have no known competing financial interests or personal relationships that could have appeared to influence the work reported in this paper.
